# ﻿A new species of the genus *Leptobrachella* (Anura, Megophryidae) from central Guangdong Province, China

**DOI:** 10.3897/zookeys.1259.164646

**Published:** 2025-11-11

**Authors:** Qi-Qi Zhang, Shi-Shi Lin, Yuan-Hang Li, Hong-Lin Su, Hong-Hui Chen, Xiu-Yu Zhang, Zhao-Chi Zeng, Jian Wang

**Affiliations:** 1 Guangdong Polytechnic of Environmental Protection Engineering, Foshan 528216, China Guangdong Polytechnic of Environmental Protection Engineering Foshan China; 2 Guangdong Provincial Academy of Environmental Science, Guangzhou 510555, China Guangdong Provincial Academy of Environmental Science Guangzhou China

**Keywords:** *Leptobrachella kungfu* sp. nov., *Leptobrachella yunkaiensis*, molecular phylogeny, morphology, regional conservation, taxonomy

## Abstract

In this study, a new species, *Leptobrachella kungfu***sp. nov.**, from Foshan City, central Guangdong Province, China, is described based on a combination of molecular and morphological data. Phylogenetically, the new species is closely related to *L. yunkaiensis* from western Guangdong Province and can be distinguished from all congeners by a combination of discrete morphological characters. A key to the species of this genus from Guangdong and a distribution map are provided to support further regional research and conservation.

## ﻿Introduction

The genus *Leptobrachella* Smith, 1925 represents one of the most species-rich groups of frogs with more than 110 recognized species. Species of this genus are forest dwellers that are widely distributed in southern China, northeastern India, and Myanmar through the mainland region of Southeast Asia to peninsular Malaysia and the island of Borneo (Frost 2025). Morphological conservativeness hinders our understanding of the true diversity of this group (Wang et al. 2019; Wang et al. 2022) and rapid discovery of many new species within this genus emphasizes the importance of regional research. The taxonomic status of the populations in Guangdong Province, China, was reviewed by Lin et al. (2022), which provides a distribution map and a key to six species.

**Figure 1.**
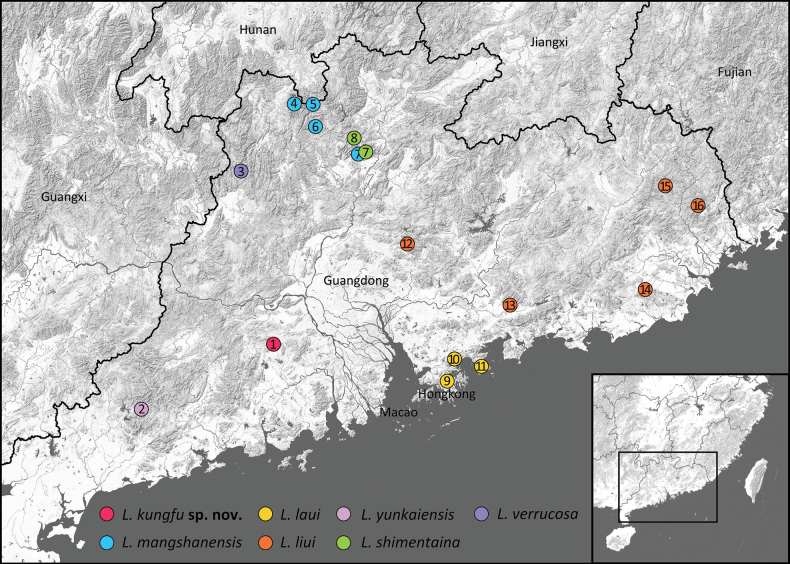
Localities of *Leptobrachella* species occurring in Guangdong Province and Hong Kong, China: *Leptobrachella kungfu* sp. nov. (site ID 1, Mt Laoxiangshan, Gaoming District); *L. yunkaiensis* (site ID 2, Mt. Yunkai, Xinyi city); *L. verrucosa* (site ID 3, Lianshan Bijiashan Nature Reserve, Lianshan county); *L. mangshanensis* (site ID 4, Mt. Dadong, Lianxian county; site ID 5, Nanling Nature Reserve, Ruyuan county; site ID 6, Mt. Tianjing, Ruyuan county; site ID 7, Shimentai Nature Reserve, Yingde city); *L. shimentaina* (site ID 7, Shimentai Nature Reserve, Yingde city; site ID 8, Luokeng Nature Reserve, Qujiang district); *L. laui* (site ID 9, Tai Mo Shan, Hong Kong; site ID 10, Mt Wutong, Shenzhen city; site ID 11, Mt Paiya, Shenzhen city); *L. liui* (site ID 12, Mt Nankun, Longmen county; site ID 13, Gutian Nature Reserve, Huidong county; site ID 14, Mt Motianshi, Puning city; site ID 15, Mt Tongguzhang, Fengshun county; site ID 16, Mt Fenghuang, Chaozhou city).

During recent field surveys in the mountainous areas of Foshan City, Guangdong, China (Fig. 1), we collected six adult male specimens of *Leptobrachella*. Preliminary morphological examination indicated that they could be distinguished from recognized congeners by a series of discrete features. Subsequent molecular analysis further revealed that these specimens represent a separate evolutionary lineage. Considering both the morphological differences and their genetic divergences, these specimens are described herein as a new species.

## ﻿Materials and methods

### ﻿Phylogenetic analyses

A total of 110 samples, including 96 *Leptobrachella* species and two outgroup samples were used in this study, encompassing six newly collected individuals and others downloaded from GenBank. Detailed information for all samples is given in Suppl. material 1: table S1. For the newly collected samples, genomic DNA was extracted using the TIANamp Genomic DNA Kit (Tiangen Biotech Co., Ltd., Beijing). The 16S ribosomal RNA mitochondrial gene (16S rRNA) was used for phylogenetic analyses with the primer, PCR experimental procedures, and sequencing approach following Wang et al. (2020). Sequences were aligned using Clustal X 2.0 (Thompson et al. 1997) with default parameters and a final dataset of 512 bp was obtained for further analysis. Jmodeltest v. 2.1.2 (Darriba et al. 2012) was used to obtain the best-fitting nucleotide substitution model with Akaike and Bayesian information criteria, and GTR + I + G was selected as the best-fit model. Phylogenetic analysis was conducted using Bayesian inference (BI) in MrBayes 3.2.4 (Ronquist et al. 2012) and maximum likelihood (ML) in RaxML (Stamatakis 2006) with RAxML GUI 1.3 (Silvestro and Michalak 2012). For the ML analysis, an optimal tree was obtained, and branch support was evaluated with 1000 rapid bootstrap replicates. For the BI analysis, two independent runs with four Markov Chain Monte Carlo simulations were performed for ten million iterations and sampled every 1000 iterations. The first 25% of the samples were discarded as burn-in, leaving 7500 samples in the final summary. Convergence of the Markov Chain Monte Carlo simulations was assessed by PSRF < 0.01 and ESS (effective sample size) value > 200 using Tracer 1.4 (http://tree.bio.ed.ac.uk/software/tracer/). Nodes having ML bootstrap values (BS) ≥ 70 and BI posterior probabilities (BPP) ≥ 0.90 were considered well supported. Mean genetic distances among all *Leptobrachella* samples were calculated in MEGA 11 (Tamura et al. 2021) using the uncorrected *p*-distance model.

### ﻿Morphological examination

Following Fei et al. (2009) and Rowley et al. (2013), measurements were taken with digital calipers from preserved specimens (Neiko 01407A Stainless Steel 6-Inch Digital Calipers) to the nearest 0.1 mm. Measurements include: snout-vent length (**SVL**) from the tip of the snout to the posterior margin of the vent; head length (**HDL**) from the tip of the snout to the articulation of the jaw; head width (**HDW**) at the commissure of the jaws; snout length (**SNT**) from the tip of the snout to the anterior corner of the eye; eye diameter (**EYE**) from the anterior corner of the eye to posterior corner of the eye; internasal distance (**IND**); interorbital distance (**IOD**); horizontal diameter of tympanum (**TMP**); tympanum-eye distance (**TEY**) from the anterior edge of the tympanum to the posterior corner of the eye; tibial length (**TIB**) from the outer surface of the flexed knee to the heel; manus length (**ML**) from the tip of the third finger to the inner palmar tubercle proximal edge; pes length (**PE**) from the tip of the fourth toe to the inner metatarsal tubercle proximal edge; length of lower arm and hand (**LAHL**) from the tip of the third finger to the elbow; and hindlimb length (**HLL**) from the tip of the fourth toe to the vent.

Sex was determined by direct observation of calling in life. Comparative morphological data for other congeneric species of *Leptobrachella* were obtained from museum specimens (Appendix 1) and from the literature (Table 1). All newly collected specimens were euthanized with MS 222, then fixed in 10% buffered formalin, then later transferred to 70% ethanol for preservation. Abbreviations for museums and institutes include Guangdong Polytechnic of Environmental Protection Engineering, Foshan, China **(GEP)**, and The Museum of Biology, Sun Yat-sen University, Guangzhou, China**(SYS).**

**Table 1.** Data sources of the currently known species of the genus *Leptobrachella*.

ID	Leptobrachella species	Literature
1	*L. aerea* (Rowley, Stuart, Richards, Phimmachak & Sivongxay, 2010c)	Rowley et al. 2010c
2	*L. albomarginata* Wu, Yu, Kilunda, Murphy & Che, 2025	Wu et al. 2025
3	*L. aspera* Wang, Lyu, Qi & Wang, 2020	Wang et al. 2020
4	*L. alpina* (Fei, Ye & Li, 1990)	Fei et al. 1990, 2009, 2016
5	*L. applebyi* (Rowley & Cao, 2009)	Rowley and Cao 2009
6	*L. arayai* (Matsui, 1997)	Matsui 1997
7	*L. ardens* (Rowley, Tran, Le, Dau, Peloso, Nguyen, Hoang, Nguyen & Ziegler, 2016)	Rowley et al. 2016
8	*L. aurantirosea* Ninh, Nguyen, Le, Nguyen, Quoc, Orlov, Bezman-Moseyko, Le, Nguyen & Ziegler, 2024	Ninh et al. 2024
9	*L. batxatensis* Hoang, Pham, Phan, Do, Wang, Jiang & Nguyen, 2025	Hoang et al. (2025)
10	*L. baluensis* Smith, 1931	Dring 1983; Eto et al. 2016
11	*L. bashaensis* Lyu, Dai, Wei, He, Yuan, Shi, Zhou, Ran, Kuang, Guo, Wei & Yuan, 2020	Lyu et al. 2020
12	*L. bijie* Wang, Li, Li, Chen & Wang, 2019	Wang et al. 2019, 2021, 2022
13	*L. bidoupensis* (Rowley, Le, Tran & Hoang, 2011)	Rowley et al. 2011
14	*L. bondangensis* Eto, Matsui, Hamidy, Munir & Iskandar, 2018	Eto et al. 2018
15	*L. botsfordi* (Rowley, Dau & Nguyen, 2013)	Rowley et al. 2013
16	*L. bourreti* (Dubois, 1983)	Ohler et al. 2011
17	*L. brevicrus* Dring, 1983	Dring 1983; Eto et al. 2015
18	*L. crocea* (Rowley, Hoang, Le, Dau & Cao, 2010)	Rowley et al. 2010a
19	*L. chishuiensis* Li, Liu, Wei & Wang, 2020	Li et al. 2020
20	*L. damingshanensis* Chen, Yu, Cheng, Meng, Wei, Zhou & Lu, 2021	Chen et al. 2021b
21	*L. dayaoshanensis* Chen, Yu, Meng & Qin, 2024	Chen et al. 2024
22	*L. dong* Liu, Shi, Li, Zhang, Xiang, Wei & Wang, 2023	Liu et al. 2023
23	*L. dorsospina* Wang, Lyu, Qi & Wang, 2020	Wang et al. 2020
24	*L. dringi* (Dubois, 1987)	Inger et al. 1995; Matsui and Dehling 2012
25	*L. dushanensis* Li, Li, Cheng, Liu, Wei & Wang, 2024	Li et al. 2024
26	*L. duyenae* Hoang, Pham, Phan, Do, Wang, Jiang & Nguyen, 2025	Hoang et al. 2025
27	*L. eos* (Ohler, Wollenberg, Grosjean, Hendrix, Vences, Ziegler & Dubois, 2011)	Ohler et al. 2011
28	*L. feii* Chen, Yuan & Che, 2020	Chen et al. 2020
29	*L. firthi* (Rowley, Hoang, Dau, Le & Cao, 2012)	Rowley et al. 2012
30	*L. fritinniens* (Dehling & Matsui, 2013)	Dehling and Matsui 2013
31	*L. fuliginosa* (Matsui, 2006)	Matsui 2006
32	*L. flaviglandulosa* Chen, Wang & Che, 2020	Chen et al. 2020
33	*L. fusca* Eto, Matsui, Hamidy, Munir & Iskandar, 2018	Eto et al. 2018
34	*L. gracilis* (Günther, 1872)	Günther 1872; Dehling 2012b
35	*L. graminicola* Nguyen, Tapley, Nguyen, Luong & Rowley, 2021	Nguyen et al. 2021
36	*L. guinanensis* Chen, Li, Peng & Liu, 2024	Chen et al. 2024
37	*L. hamidi* (Matsui, 1997)	Matsui 1997
38	*L. heteropus* (Boulenger, 1900)	Boulenger 1900
39	*L. huynhi* Hoang, Luong, Nguyen, Nguyen, Ninh, Le, Ziegler & Pham, 2024	Hoang et al. 2024
40	*L. isos* (Rowley, Stuart, Neang, Hoang, Dau, Nguyen & Emmett, 2015)	Rowley et al. 2015a
41	*L. itiokai* Eto, Matsui & Nishikawa, 2016	Eto et al. 2016
42	*L. juliandringi* Eto, Matsui & Nishikawa, 2015	Eto et al. 2015
43	*L. jinshaensis* Cheng, Shi, Li, Liu, Li & Wang, 2021	Cheng et al. 2021
44	*L. jinyunensis* Shi, Shen, Wang, Jiang & Wang, 2023	Shi et al. 2023
45	*L. kajangensis* (Grismer, Grismer & Youmans, 2004)	Grismer et al. 2004
46	*L. kalonensis* (Rowley, Tran, Le, Dau, Peloso, Nguyen, Hoang, Nguyen & Ziegler, 2016)	Rowley et al. 2016
47	*L. kecil* (Matsui, Belabut, Ahmad & Yong, 2009)	Matsui et al. 2009
48	*L. khasiorum* (Das, Tron, Rangad & Hooroo, 2010)	Das et al. 2010
49	*L. korifi* Matsui, Panha & Eto, 2023	Matsui et al. 2023
50	*L. lateralis* (Anderson, 1871)	Anderson 1871; Humtsoe et al. 2008
51	*L. laui* (Sung, Yang & Wang, 2014)	Sung et al. 2014
52	*L. liui* (Fei & Ye, 1990)	Fei et al. 1990, 2009; Sung et al. 2014; Wang et al. 2022
53	*L. macrops* (Duong, Do, Ngo, Nguyen & Poyarkov, 2018)	Duong et al. 2018
54	*L. maculosa* (Rowley, Tran, Le, Dau, Peloso, Nguyen, Hoang, Nguyen & Ziegler, 2016)	Rowley et al. 2016
55	*L. mangshanensis* (Hou, Zhang, Hu, Li, Shi, Chen, Mo & Wang, 2018)	Hou et al. 2018; Wang et al. 2022
56	*L. maoershanensis* (Yuan, Sun, Chen, Rowley & Che, 2017)	Yuan et al. 2017
57	*L. marmorata* (Matsui, Zainudin & Nishikawa, 2014b)	Matsui et al. 2014b
58	*L. maura* (Inger, Lakim, Biun & Yambun, 1997)	Inger et al. 1997
59	*L. melanoleuca* (Matsui, 2006)	Matsui 2006
60	*L. melica* (Rowley, Stuart, Neang & Emmett, 2010)	Rowley et al. 2010b
61	*L. minima* (Taylor, 1962)	Taylor 1962; Ohler et al. 2011
62	*L. mjobergi* Smith, 1925	Eto et al. 2015
63	*L. murphyi* Chen, Suwannapoom, Wu, Poyarkov, Xu, Pawangkhanant & Che, 2021	Chen et al. 2021a
64	*L. nahangensis* (Lathrop, Murphy, Orlov & Ho, 1998)	Lathrop et al. 1998
65	*L. natunae* (Günther, 1895)	Günther 1895
66	*L. namdongensis* Hoang, Nguyen, Luu, Nguyen & Jiang, 2019	Hoang et al. 2019
67	*L. neangi* Stuart & Rowley, 2020	Stuart and Rowley 2020
68	*L. niveimontis* Chen, Poyarkov, Yuan & Che, 2020	Chen et al. 2020
69	*L. nokrekensis* (Mathew & Sen, 2010)	Mathew and Sen 2010
70	*L. nyx* (Ohler, Wollenberg, Grosjean, Hendrix, Vences, Ziegler & Dubois, 2011)	Ohler et al. 2011
71	*L. oshanensis* (Liu, 1950)	Liu, 1950; Shi et al. 2021
72	*L. pallida* (Rowley, Tran, Le, Dau, Peloso, Nguyen, Hoang, Nguyen & Ziegler, 2016)	Rowley et al. 2016
73	*L. palmata* Inger & Stuebing, 1992	Inger and Stuebing 1992
74	*L. parva* Dring, 1983	Dring 1983
75	*L. pelodytoides* (Boulenger, 1893)	Boulenger 1893; Ohler et al. 2011
76	*L. petrops* (Rowley, Dau, Hoang, Le, Cutajar & Nguyen, 2017)	Rowley et al. 2017a
77	*L. phiadenensis* Luong, Hoang, Pham, Ziegler & Nguyen, 2023	Luong et al. 2023
78	*L. phiaoacensis* Luong, Hoang, Pham, Ziegler & Nguyen, 2023	Luong et al. 2023
79	*L. pingbianensis* (Rao 2020)	Rao 2020
80	*L. picta* (Malkmus, 1992)	Malkmus 1992
81	*L. platycephala* (Dehling, 2012)	Dehling 2012a
82	*L. pluvialis* (Ohler, Marquis, Swan & Grosjean, 2000)	Ohler et al. 2000, 2011
83	*L. puhoatensis* (Rowley, Dau & Cao, 2017)	Rowley et al. 2017b
84	*L. purpurus* (Yang, Zeng & Wang, 2018)	Yang et al. 2018
85	*L. purpuraventra* Wang, Li, Li, Chen & Wang, 2019	Wang et al. 2019
86	*L. pyrrhops* (Poyarkov, Rowley, Gogoleva, Vassilieva, Galoyan & Orlov, 2015)	Poyarkov et al. 2015
87	*L. rowleyae* (Nguyen, Poyarkov, Le, Vo, Ninh, Duong, Murphy & Sang, 2018)	Nguyen et al. 2018
88	*L. sabahmontana* (Matsui, Nishikawa & Yambun, 2014)	Matsui et al. 2014a
89	*L. serasanae* Dring, 1983	Dring 1983
90	*L. shangsiensis* Chen, Liao, Zhou & Mo, 2019	Chen et al. 2019
91	*L. shimentaina* Wang, Lyu & Wang, 2022	Wang et al. 2022
92	*L. sinorensis* Matsui, Panha & Eto, 2023	Matsui et al. 2023
93	*L. sola* (Matsui, 2006)	Matsui 2006
94	*L. suiyangensis* Luo, Xiao, Gao & Zhou, 2020	Luo et al. 2020
95	*L. sungi* (Lathrop, Murphy, Orlov & Ho, 1998)	Lathrop et al. 1998
96	*L. shiwandashanensis* Chen, Peng, Pan, Liao, Liu & Huang, 2021	Chen et al. 2021c
97	*L. tadungensis* (Rowley, Tran, Le, Dau, Peloso, Nguyen, Hoang, Nguyen & Ziegler, 2016)	Rowley et al. 2016
98	*L. tamdil* (Sengupta, Sailo, Lalremsanga, Das & Das, 2010)	Sengupta et al. 2010
99	*L. tengchongensis* (Yang, Wang, Chen & Rao, 2016)	Yang et al. 2016
100	*L. tuberosa* (Inger, Orlov & Darevsky, 1999)	Inger et al. 1999
101	*L. ventripunctata* (Fei, Ye & Li, 1990)	Fei et al. 1990, 2009, 2016
102	*L. verrucosa* Wang, Zeng, Lin & Li, 2022	Lin et al. 2022
103	*L. wuhuangmontis* Wang, Yang & Wang, 2018	Wang et al. 2018
104	*L. wulingensis* Qian, Xia, Cao, Xiao & Yang, 2020	Qian et al. 2020
105	*L. wumingensis* Chen, Peng, Li & Yu, 2023	Chen et al. 2023
106	*L. xishuiensis* Luo, Deng & Zhou, 2025	Luo et al. 2025
107	*L. yingjiangensis* (Yang, Zeng & Wang, 2018)	Yang et al. 2018
108	*L. yongshunensis* Huang, Wu, Jiang & Zhang, 2025	Huang et al. 2025
109	*L. yunkaiensis* Wang, Li, Lyu & Wang, 2018	Wang et al. 2018
110	*L. yeae* Shi, Hou, Song, Jiang & Wang, 2021	Shi et al. 2021
111	*L. yunyangensis* Luo, Deng & Zhou, 2022	Luo et al. 2022
112	*L. zhangyapingi* (Jiang, Yan, Suwannapoom, Chomdej & Che, 2013)	Jiang et al. 2013

## ﻿Results

**Figure 2.**
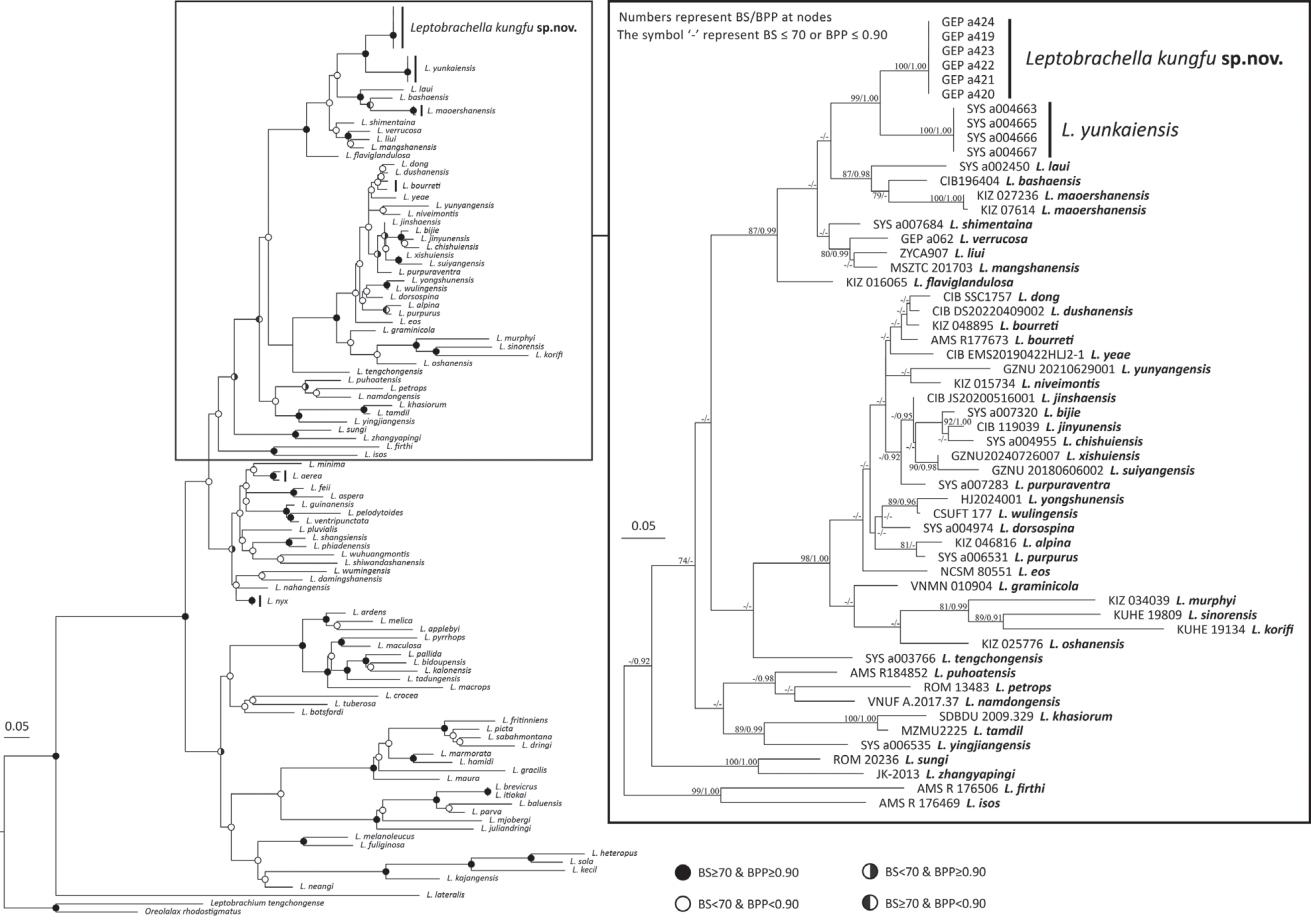
Phylogeny results from a Maximum Likelihood analysis based on partial DNA sequences of the mitochondrial 16S r RNA gene. Voucher before species name at the branch terminal corresponds to the voucher ID in the Suppl. material 1: table S1.

In the phylogenetic analyses, both the ML and Bayesian approaches yielded identical topologies, and all *Leptobrachella* samples formed a monophyletic clade with strong support (Fig. 2). The interspecific uncorrected mean *p*-distance of *Leptobrachella* (Suppl. material 2: table S2) ranged from 0.7% (between *L. bijie* and *L. jinyunensis*) to 29.6% (between *L. kecil* and *L. lateralis*). The *Leptobrachella* samples from Mt. Laoxiangshan, Foshan form a monophyletic lineage with strong support (BS = 100; BPP = 1.00). This lineage forms a sister clade to *L. yunkaiensis* with strong support (BS = 99; BPP = 1.00) and exhibits a significant mean *p*-distance of 4.9% in 16S rRNA gene. Moreover, detailed morphological examination revealed discrete, diagnostic (non-overlapping ranges in traditional characters) differences among the specimens from this independent lineage and all other congeners. Therefore, both phylogenetic results and morphological comparisons support that the lineage from Mt Laoxiangshan represent an undescribed new species, and we describe this new species below.

### ﻿Taxonomic account

#### ﻿ Leptobrachella kungfu sp. nov.

Taxon classificationAnimaliaAnuraMegophryidae

4680A168-394C-5C95-8F87-A7B40C9723EE

https://zoobank.org/1D4A6B13-3696-431B-B214-20757AD0B5AB

**Figure 3.**
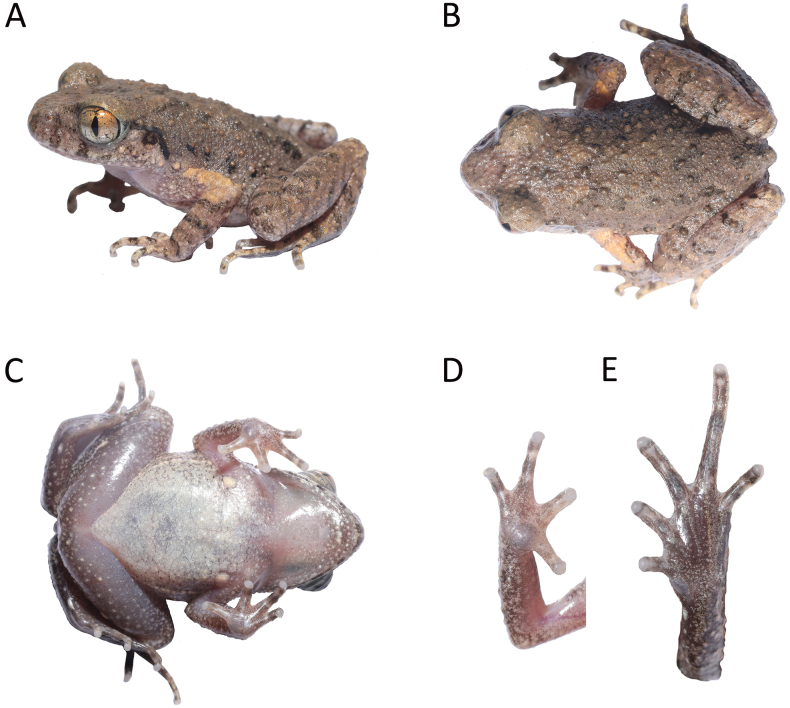
Morphological features of the holotype of *Leptobrachella kungfu* sp. nov., GEP a419 in life: A. Lateral view; B. Dorsal view; C. Ventral view; D. Ventral view of hand; E. Ventral view of foot.

Fig. 3 Foshan Leaf Litter Toad (in English) / Fo Shan Zhang Tu Chan (佛山掌突蟾 in Chinese)

##### Type material.

***Holotype*** • ♂. GEP a419, collected by Shi-Shi Lin and Yuan-Hang Li on 29 April 2025 from Mt Laoxiangshan / 老香山 (22°46'50.63"N, 112°26'37.95"E; ca. 250 m a.s.l.), Gaoming District, Foshan City, Guangdong Province, **China**. ***Paratypes*.** • 5 ♂: GEP a420–423, SYS a009517 (field number: GEP a424), the same collection data as the holotype.

##### Diagnosis.

(1) Small body size [SVL 25.7–28.2 mm in six adult males]; (2) iris bicolored, upper half coppery orange and lower half grayish brown; (3) tympanum distinct; (4) distinct black supratympanic line; (5) fingers without lateral fringes; (6) toes with rudimentary webbing and wide lateral fringes; (7) longitudinal ridges under toes continuous; (8) heels just meeting when adpressed, tibial-tarsal articulation reaching loreal region; (9) dorsal surface shagreened with dense tubercles and raised warts, lacking skin ridges; (10) ventral surface smooth; (11) dorsum grayish brown to dark brown with indistinct dark brown scattered markings; (12) flank with several dark spots; (13) surface of throat, chest and abdomen creamy white.

##### Description of holotype.

Adult male. Body size small, SVL 25.8 mm. Head length slightly longer than head width, HDW/HDL 0.92; snout slightly protruding, projecting slightly beyond margin of lower jaw; nostril closer to snout than eye; canthus rostralis gently rounded; loreal region slightly concave; interorbital space flat, internarial distance slightly larger than interorbital distance, IND/IOD 1.03; pineal ocellus absent; pupil vertical; eye diameter slightly longer than snout, SNT/EYE 0.97; tympanum distinct, rounded, diameter smaller than that of eye and larger than tympanum-eye distance, TMP/EYE 0.50, TEY/TMP 0.56; upper margin of tympanum in contact with supratympanic ridge; vomerine teeth absent; a single vocal sac; vocal sac openings slit-like, paired, located posterolaterally on floor of mouth, close to margins of mandible; tongue deeply notched distally; supratympanic ridge distinct, extending from posterior corner of eye to supra-axillary gland.

Tips of fingers rounded, slightly swollen; relative finger lengths I = II = IV < III; nuptial pad absent; subarticular tubercles absent; inner palmar tubercle large, rounded, distinctly separated from outer palmar tubercle; outer palmar tubercle small, rounded; fingers lacking interdigital webbing and lateral fringes. Tips of toes rounded, slightly swollen; relative toe length I < II < V < III < IV; longitudinal ridges under toes continuous; inner metatarsal tubercle large, oval; outer metatarsal tubercle absent; interdigital webbing between toes rudimentary; wide lateral fringes present on all toes. Tibia 49% of snout-vent length; tibiotarsal articulation reaches loreal region; heels just meeting where thighs are appressed at right angles with respect to body.

Skin on dorsum shagreened and scattered with dense tubercles and raised warts; ventral skin smooth; pectoral and femoral glands oval, both larger in diameter than tip of digits; femoral gland situated on posteroventral surface of thigh, closer to knee than to vent; supra-axillary gland raised. Ventrolateral gland distinctly visible, forming a longitudinal discontinuous series.

##### Coloration in life.

Dorsal background color grayish brown with indistinct dark brown markings and rounded spots. A dark brown inverted triangular marking present in the interorbital region, connected to dark brown W-shaped marking on occipital region. Tympanum dark brown, lower margin light yellow. Supratympanic line black. A pair of dark brown vertical bars under the eyes; transverse dark brown bars on dorsal surfaces of distal limbs and digits. Distinct dark brown blotches on flanks; surfaces of elbows and upper arms coppery orange.

Surface of throat, chest, and belly creamy white. Undersides of limbs grayish purple with numerous white spots. Supra-axillary, pectoral, femoral and ventrolateral glands light yellow. Iris bicolored: upper half coppery orange, lower half grayish brown.

##### Coloration in preservative.

Dorsal background color dark brown, scattered with irregular gray pigmentations. All markings, bars, and spots indistinct. Tympanum dark brown.

Ventral surface grayish white; grayish white spots absent, dark brown spots become more distinct. Supra-axillary, pectoral, and ventrolateral glands greyish white.

##### Variation.

Measurements and body proportions are listed in Table 2. All the specimens showed relatively small differences in coloration and color patterns.

**Table 2.** Measurements (minimum–maximum (mean ± SD); in mm), and body proportions of the type specimens of *Leptobrachella kungfu* sp. nov.

Voucher	GEP a419	GEP a420	GEP a421	GEP a422	GEP a423	SYS a009517	Range
Sex	Male	Male	Male	Male	Male	Male	Males (*n* = 6)
SVL	25.8	26.6	28.2	25.7	25.7	26.8	25.7–28.2 (26.5 ± 1.0)
HDL	10.4	10.9	10.6	10.3	10.8	11.0	10.3–11.0 (10.6 ± 0.3)
HDW	9.6	10.6	10.2	10.1	10.2	10.3	9.6–10.6 (10.2 ± 0.3)
SNT	3.9	4.2	4.2	4.3	4.4	4.5	3.9–4.5 (4.2 ± 0.2)
IND	3.1	2.9	2.9	3.2	2.9	3.1	2.9–3.2 (3.0 ± 0.1)
IOD	3.0	3.0	2.8	2.9	3.2	3.1	2.8–3.2 (3.0 ± 0.2)
EYE	4.0	3.9	4.2	3.6	4.1	4.0	3.6–4.2 (4.0 ± 0.2)
TMP	2.0	2.0	1.8	2.0	2.1	1.9	1.8–2.1 (2.0 ± 0.1)
TEY	1.1	1.1	1.0	0.9	1.0	0.9	0.9–1.1 (1.0 ± 0.1)
ML	6.6	6.9	7.6	7.1	6.8	6.8	6.6–7.6 (7.0 ± 0.3)
LAHL	12.5	13.6	14.2	12.7	12.8	12.4	12.4–14.2 (13.0 ± 0.7)
PL	12.1	12.7	13.4	11.8	11.9	11.7	11.7–13.4 (12.3 ± 0.6)
TIB	12.5	13.2	13.5	12.3	12.5	12.4	12.3–13.4 (12.7 ± 0.5)
HLL	43.7	43.8	46.3	44.0	43.9	41.6	41.6–46.3 (43.9 ± 1.5)

##### Etymology.

The type locality of the new species, Foshan, is known as the City of Chinese Kungfu. Many renowned Kungfu masters, such as Fei-Hong Huang (黄飞鸿), Zan Liang (梁赞), Yip Man (= Wen Ye / 叶问), and Bruce Lee (= Xiao-Long Li / 李小龙), had ancestral homes in Foshan and received their training there. The specific name *kungfu* is chosen to commemorate an important cultural aspect of Foshan City.

##### Distribution, ecology, and conservation.

*Leptobrachella kungfu* sp. nov. is currently known only from its type locality (Fig. 1), ca 800–1400 m a.s.l. The new species inhabits clear-water rocky streams in primary forests surrounded by broad-leaved forest on granite landforms. Males were observed calling while perched on rocks or on the leaves of dwarf shrubs near flowing seeps. The breeding season lasts from February to June according to our long-term observation. Given that the current information on the distribution and threats of this species is still not fully understood, we recommend the new species be listed as Data Deficient (DD) according to the International Union for Conservation of Nature (IUCN) Red List categories and criteria.

##### Comparisons.

In the phylogenetic tree (Fig. 2), *Leptobrachella kungfu* sp. nov. is most closely related to *L. yunkaiensis*. However, the new species differs from the latter by the absence of skin ridges on the dorsal surface (vs present), the absence of lateral fringes on the fingers (vs present), the absence of dark brown speckling on the belly (vs present), surface of belly cream white (vs pinkish), black supratympanic line distinct (vs weak).

*Leptobrachella kungfu* sp. nov. differs from the other five species occurring in Guangdong Province, i.e. *L. laui*, *L. liui*, *L. mangshanensis*, *L. shimentaina*, and *L. verrucosa*, by the absence of lateral fringes on the fingers (vs present in *L. laui* and males of *L. shimentaina*), the absence of skin ridges on the dorsal surface (vs present in *L. liui*, *L. mangshanensis*, and *L. shimentaina*), the presence of wide lateral fringes on the toes (vs presence of narrow lateral fringes on toes in *L. mangshanensis* and *L. verrucosa*), longitudinal ridges under toes continuous (vs longitudinal ridges interrupted in *L. liui*). *Leptobrachella kungfu* sp. nov. further differs from its closely related species, i.e. *L. bashaensis*, *L. maoershanensis*, and *L. flaviglandulosa*, by the presence of wide lateral fringes on the toes (vs narrow lateral fringes).

Compared with the 26 known congeners of the genus *Leptobrachella* occurring south of the Isthmus of Kra, by the presence of supra-axillary and ventrolateral glands, *L. kungfu* sp. nov. can easily be distinguished from *L. arayai*, *L. dringi*, *L. fritinniens*, *L. gracilis*, *L. hamidi*, *L. heteropus*, *L. kajangensis*, *L. kecil*, *L. marmorata*, *L. melanoleuca*, *L. maura*, *L. picta*, *L. platycephala*, *L. sabahmontana*, and *L. sola*, all of which lack the supra-axillary and ventrolateral glands; and by its distinctly larger male body size, SVL 25.7–28.2 mm, *L. kungfu* sp. nov. differs from the smaller *L. baluensis* (SVL 14.9–15.9 mm), *L. brevicrus* (SVL 17.1–17.8 mm), *L. bondangensis* (SVL 17.8 mm), *L. fusca* (SVL 16.3 mm), *L. itiokai* (SVL 15.2–16.7 mm), *L. juliandringi* (SVL 17.0–17.2 mm), *L. mjobergi* (SVL 15.7–19.0 mm), *L. natunae* (SVL 17.6 mm), *L. parva* (SVL 15.0–16.9 mm), and *L. palmata* (SVL 14.4–16.8 mm); and is even distinctly larger than female *L. serasanae* (SVL 16.9 mm).

In having black spots on the flanks, *Leptobrachella kungfu* sp. nov. further differs from *L. aerea*, *L. botsfordi*, *L. crocea*, *L. eos*, *L. firthi*, *L. isos*, *L. pallida*, *L. petrops*, and *L. tuberosa*, all of which lack black spots on the flanks.

For the remaining 71 members of the genus *Leptobrachella*, males of the new species (SVL 25.7–28.2 mm) differs from males of the larger *L. nahangensis* (40.8 mm), *L. sungi* (48.3–52.7 mm), and *L. zhangyapingi* (48.5–52.5 mm). By the possession of toes with rudimentary webbing and wide lateral fringes, the new species can be distinguished from *L. aurantirosea*, *L. ardens*, *L. batxatensis*, *L. duyenae*, *L. huynhi*, *L. kalonensis*, *L. maculosa*, *L. neangi*, *L. rowleyae*, *L. shiwandashanensis*, *L. tadungensis*, and *L. xishuiensis* (no webbing); *L. pelodytoides*, and *L. tamdil* (wide webbing); *L. applebyi*, *L. lateralis*, *L. macrops*, *L. melica*, *L. minima*, *L. nyx*, *L. oshanensis*, *L. pluvialis*, *L. pyrrhops*, *L. ventripunctata*, and *L. wumingensis* (no lateral fringes); *L. albomarginata*, *L. aspera*, *L. bidoupensis*, *L. bijie*, *L. bourreti*, *L. chishuiensis*, *L. damingshanensis*, *L. dorsospina*, *L. feii*, *L. fuliginosa*, *L. jinshaensis*, *L. jinyunensis*, *L. korifi*, *L. niveimontis*, *L. puhoatensis*, *L. phiaoacensis*, *L. phiadenensis*, *L. purpuraventra*, *L. sinorensis*, *L. shangsiensis*, *L. suiyangensis*, *L. tengchongensis*, *L. wuhuangmontis*, *L. wulingensis*, *L. yongshunensis*, *L. yeae*, and *L. yunyangensis* (narrow lateral fringes). By the dorsum not having any skin ridges, the new species can be distinguished from *L. alpina*, *L. dayaoshanensis*, *L. dong*, *L. dushanensis*, *L. guinanensis*, *L. nokrekensis*, *L. murphyi*, and *L. pingbianensis* (dorsum with skin ridges); *L. khasiorum* (dorsum with isolated, scattered tubercles), *L. graminicola*, *L. namdongensis*, *L. purpurus*, and *L. yingjiangensis* (dorsum lacking enlarged warts).

## ﻿Discussion

The new species, *Leptobrachella kungfu* from central Guangdong, is a sister taxon to *L. yunkaiensis* from western Guangdong, China, with strong support (BS = 99; BPP = 1.00) and significant divergence (with mean *p*-distance of 4.9% in 16S rRNA gene), and exhibits a unique morphological apomorphy. However, these two species currently only have localized distribution ranges. Thus, studies on their distribution patterns, historical diffusion paths, ecological niches, and adaptability are worthy of in-depth attention.

The rapid development and application of molecular biology techniques in taxonomic research have led to significant advances in the discovery of new species and in the classification of superspecies across many biological groups with evolutionary histories (Lyu et al. 2023). The genus *Leptobrachella* is one of the major groups within the family Megophryidae, which is rich in species diversity and widely distributed. As with other megophryid genera, studies of taxonomy and species diversity of *Leptobrachella* have been challenged by morphological conservativeness among the majority of species (Wang et al. 2022). Therefore, accurate descriptions of each species using traditional morphological traits remains of great significance for species identification and conservation. This highlights the need for gradually compiling provincial reviews of these frogs.

Below, an updated key for all species within the genus *Leptobrachella* occurring in Guangdong Province is provided, which includes *L. kungfu*, *L. laui*, *L. liui*, *L. mangshanensis*, *L. shimentaina*, *L. yunkaiensis*, and *L. verrucosa*.

### ﻿Key to *Leptobrachella* species occurring in Guangdong Province, China

**Table:** 

1	Longitudinal ridges under toes interrupted	***L. liui***
–	Longitudinal ridges under toes continuous	**2**
2	Toes with narrow lateral fringes in males	**3**
–	Toes with wide lateral fringes in males	**4**
3	Dorsal skin almost smooth with tiny transparent spines, small tubercles, and sparse short skin ridges	***L. mangshanensis***
–	Dorsal skin shagreened with numerous conical tubercles, lacking spines, enlarged warts or skin ridges	***L. verrucosa***
4	Fingers with lateral fringes	**5**
–	Fingers without lateral fringes	***L. kungfu***
5	Dorsal skin without ridges, ventral coloration white	***L. laui***
–	Dorsal skin with ridges, ventral coloration pink	**6**
6	Dorsal skin shagreened with rounded granular tubercles, longitudinal ridges under toes with constrictions at interphalangeal articulations	***L. shimentaina***
-	Dorsal skin shagreened with raised and enlarged warts, longitudinal ridges under toes without constrictions at interphalangeal articulations	***L. yunkaiensis***

## Supplementary Material

XML Treatment for Leptobrachella kungfu

## ﻿Additional information

### Use of AI

No use of AI was reported.

### Author ORCIDs

Shi-Shi Lin https://orcid.org/0000-0001-7709-7005

Yuan-Hang Li https://orcid.org/0009-0009-6543-9245

Hong-Hui Chen https://orcid.org/0000-0001-5026-2920

Zhao-Chi Zeng https://orcid.org/0000-0003-4054-8399

Jian Wang https://orcid.org/0000-0003-4249-7767

### Data availability

All of the data that support the findings of this study are available in the main text or Supplementary Information.

## ﻿Appendix 1

Specimens examined

***Leptobrachella laui* (*n* = 26)**: China: Hong Kong: SYS a002057 (Holotype), SYS a002058; China: Guangdong: Shenzhen City: SYSa 001505–1507, 1515–1521, 3471–3472, 5644–5645.

***Leptobrachella liui* (*n* = 32)**: China: Fujian: Mt. Wuyi: SYS a001572, 1596, 2478, 2479, 5925, 5926; China: Fujian: Mt. Daiyun: SYS a001736, 6010; China: Fujian: Mt. Longqi: SYS a002505, 2506; China: Guangdong: Mt. Tongguzhang: SYS a004733–4735; China: Guangdong: Mt. Fenghuang: SYS a003698–3699; China: Guangdong: Mt. Motianshi: SYS a007610–7613; China: Guangdong: Mt. Nankun: SYS a002020, 4497; China: Guangdong: Gutian Nature Reserve: SYS a002650; China: Jiangxi: Mt. Jiulian: SYS a002104–2105; China: Jiangxi: Mt. Tongbo: SYS a001702, 2059; China: Jiangxi: Mt. Yangjifeng: SYS a006667, 6672; China: Zhejiang: Jingning: SYS a002732–2735.

***Leptobrachella mangshanensis* (*n* = 11)**: China: Hunan: Mangshan Nature Reserve: SYS a008366; China: Guangdong: Nanling Nature Reserve: SYS a002828–2830, 5754; China: Guangdong: Shimentai Nature Reserve: SYS a005763, 6880; China: Guangdong: Mt. Tianjing: SYS a002806, 2809; China: Guangdong: Mt. Dadong: SYS a002847–2848.

***Leptobrachella shimentaina* (*n* = 8)**: China: Guangdong: Yingde City: Shimentai Nature Reserve: SYS a007684 (Holotype), 4711–4712, 7685–7687, SYS a007683/CIB116079; China: Guangdong: Shaoguan City: Luokeng Nature Reserve: SYS a008329.

***Leptobrachella verrucosa* (*n* = 5)**: China: Guangdong: GEP a062 (Holotype), 059–061, 063.

***Leptobrachella yunkaiensis* (*n* = 8)**: China: Guangdong: Maoming City: Mt. Yunkai: SYS a004664/CIB107272, SYS a004663, 4665–4669, 4690.

## Supplementary materials

Supplementary material 1 Collection localities, Voucher ID, and Genbank accession numbers of the 16S rRNA for all *Leptobrachella* samples used in this study Data type docx https://binary.pensoft.net/file/1462663This dataset is made available under the Open Database License (http://opendatacommons.org/licenses/odbl/1.0/). The Open Database License (ODbL) is a license agreement intended to allow users to freely share, modify, and use this Dataset while maintaining this same freedom for others, provided that the original source and author(s) are credited.Qi-Qi Zhang, Shi-Shi Lin, Yuan-Hang Li, Hong-Lin Su, Hong-Hui Chen, Xiu-Yu Zhang, Zhao-Chi Zeng, Jian Wang

Supplementary material 2 Uncorrected mean p-distances among *Leptobrachella* species and outgroups based on the 16S rRNA fragment Data type xlsx https://binary.pensoft.net/file/1462664This dataset is made available under the Open Database License (http://opendatacommons.org/licenses/odbl/1.0/). The Open Database License (ODbL) is a license agreement intended to allow users to freely share, modify, and use this Dataset while maintaining this same freedom for others, provided that the original source and author(s) are credited.Qi-Qi Zhang, Shi-Shi Lin, Yuan-Hang Li, Hong-Lin Su, Hong-Hui Chen, Xiu-Yu Zhang, Zhao-Chi Zeng, Jian Wang
