# Supplementary material for: ﻿A new species of the genus Leptobrachella (Anura, Megophryidae) from central Guangdong Province, China
Source: Zookeys. 2025 Nov 11;1259:185–204. doi: 10.3897/zookeys.1259.164646 (PMC12627998; doi:10.3897/zookeys.1259.164646)
Supplement: Supplementary material 1 — Collection localities, Voucher ID, and Genbank accession numbers of the 16S rRNA for all Leptobrachella samples used in this study [file zookeys-1259-185_article-164646__-s001.docx]

**Table S1.** Collection localities, voucher data, and Genbank accession numbers of 16S rRNA for all *Leptobrachella* and outgroup samples used in this study.

| **NO.** | **Species** | **Voucher ID** | **Sampling Locality** | **Genbank accession number** | **Reference** |
| --- | --- | --- | --- | --- | --- |
| 1 | *Leptobrachella kungfu* **sp. nov.** | GEPa419 | Mt Laoxiangshan, Foshan, Guangdong, China | PX431625 | This study |
| 2 | *Leptobrachella kungfu* **sp. nov.** | GEPa420 | Mt Laoxiangshan, Foshan, Guangdong, China | PX431626 | This study |
| 3 | *Leptobrachella kungfu* **sp. nov.** | GEPa421 | Mt Laoxiangshan, Foshan, Guangdong, China | PX431627 | This study |
| 4 | *Leptobrachella kungfu* **sp. nov.** | GEPa422 | Mt Laoxiangshan, Foshan, Guangdong, China | PX431628 | This study |
| 5 | *Leptobrachella kungfu* **sp. nov.** | GEPa423 | Mt Laoxiangshan, Foshan, Guangdong, China | PX431629 | This study |
| 6 | *Leptobrachella kungfu* **sp. nov.** | GEPa424 | Mt Laoxiangshan, Foshan, Guangdong, China | PX431630 | This study |
| 7 | *Leptobrachella aerea* | NCSM 76038 | Vilabuly, Savannakhet, Laos | MH055809 | Chen et al. (2018) |
| 8 | *Leptobrachella aerea* | RH 60165 | Phong Nha-Ke Bang, Quang Binh, Vietnam | JN848437 | Ohler et al. (2011) |
| 9 | *Leptobrachella alpina* | KIZ 046816 | Huangcaoling, Yunnan, China | MH055866 | Chen et al. (2018) |
| 10 | *Leptobrachella applebyi* | KIZ010701 | Thua Thien-Hue, Vietnam | MH055947 | Chen et al. (2018) |
| 11 | *Leptobrachella ardens* | ZMMU-NAP-06099 | Gia Lai,Vietnam | MH055949 | Chen et al. (2018) |
| 12 | *Leptobrachella aspera* | SYS a007744 | Huanglianshan Nature Reserve, Lyuchun, Yunnan, China | MW046200 | Wang et al. (2020) |
| 13 | *Leptobrachella baluensis* | SP 21604 | Borneo, Malaysia | LC056792 | Eto et al. (2015) |
| 14 | *Leptobrachella bashaensis* | CIB196404 | Basha Nature Reserve, Guizhou, China | MW136295 | Lyu et al. (2020) |
| 15 | *Leptobrachella bidoupensis* | ZMMU-A-4797-01454 | Lam Dong, Vietnam | MH055945 | Chen et al. (2018) |
| 16 | *Leptobrachella bijie* | SYS a007320 | Zhaozishan Nature Reserve, Guizhou, China | MK414539 | Wang et al. (2019) |
| 17 | *Leptobrachella botsfordi* | AMS R176540 | Lao Cai, Vietnam | MH055953 | Chen et al. (2018) |
| 18 | *Leptobrachella bourreti* | KIZ 048895 | Xiaoqiaogou Nature Reserve, Yunnan, China | MT302638 | Chen et al. (2020) |
| 19 | *Leptobrachella bourreti* | AMS R177673 | Lao Cai, Vietnam | KR018124 | Rowley et al. (2015) |
| 20 | *Leptobrachella brevicrus* | UNIMAS 8957 | Sarawak, Malaysia | KJ831303 | Oberhummer et al. (2014) |
| 21 | *Leptobrachella chishuiensis* | SYS a004955 | Jinsha village, Chishui, Guizhou, China | OQ024818 | Shi et al. (2023) |
| 22 | *Leptobrachella crocea* | AMS R173740 | Kon Tum, Vietnam | MH055954 | Chen et al. (2018) |
| 23 | *Leptobrachella damingshanensis* | NNU 202103281 | Damingshan National Nature Reserve, Wuming County, Guangxi, China | MZ145229 | Chen et al. (2021) |
| 24 | *Leptobrachella dong* | CIB SSC1757 | Tongdao County, Hunan, China | OP764530 | Liu et al. (2023) |
| 25 | *Leptobrachella dorsospina* | SYS a004974 | Yushe Forest Park, Shuicheng, Guizhou, China | MW046197 | Wang et al. (2020) |
| 26 | *Leptobrachella dringi* | KUHE:55610 | Gunung Mulu, Malaysia | AB847553 | Matsui et al. (2014) |
| 27 | *Leptobrachella dushanensis* | CIB DS20220409002 | Dushan County, Guizhou, China | PP061389 | Li et al. (2024) |
| 28 | *Leptobrachella eos* | NCSM 80551 | Boun Tay, Phongsaly, Laos | MH055887 | Chen et al. (2018) |
| 29 | *Leptobrachella feii* | KIZ 048921 | Daweishan Nature Reserve, Yunnan, China | MH055842 | Chen et al. (2018) |
| 30 | *Leptobrachella firthi* | AMS R 176506 | Ngoc Linh Nature Reserve, Kon Tum, Vietnam | JQ739207 | Rowley et al. (2012) |
| 31 | *Leptobrachella flaviglandulosa* | KIZ 016065 | Xiaoqiaogou Nature Reserve, Yunnan, China | MT302625 | Chen et al. (2020) |
| 32 | *Leptobrachella fritinniens* | FMNH 244800 | Sabah,Malaysia | MH055971 | Chen et al. (2018) |
| 33 | *Leptobrachella fuliginosa* | KUHE 20197 | Phetchaburi, Thailand | LC201988 | Matsui et al. (2017) |
| 34 | *Leptobrachella gracilis* | FMNH 273682 | Sarawak, Malaysia | MH055972 | Chen et al. (2018) |
| 35 | *Leptobrachella graminicola* | VNMN 010904 | Mount Pu Ta Leng, Lao Cai, Vietnam | MZ224651 | Nguyen et al. (2021) |
| 36 | *Leptobrachella guinanensis* | NNU 00557 | Shangsi County, Guangxi, China | OP548561 | Chen et al. (2024) |
| 37 | *Leptobrachella hamidi* | KUHE17545 | Selangor, Malaysia | AB969286 | Matsui et al. (2014) |
| 38 | *Leptobrachella heteropus* | KUHE 15487 | Peninsula, Malaysia | AB530453 | Matsui et al. (2010) |
| 39 | *Leptobrachella isos* | AMS R 176469 | Gia Lai, Vietnam | KT824767 | Rowley et al. (2015) |
| 40 | *Leptobrachella itiokai* | KUHE:55897 | Sarawak,Malaysia | LC137805 | Eto et al. (2016) |
| 41 | *Leptobrachella jinshaensis* | CIB JS20200516001 | Lengshuihe Nature Reserve, Jinsha County, Guizhou, China | MT814014 | Cheng et al. (2021) |
| 42 | *Leptobrachella jinyunensis* | CIB 119039 | Mt.Jinyun, Beibei District, Chongqing, China | OQ024778 | Shi et al. (2023) |
| 43 | *Leptobrachella juliandringi* | KUHE 55333 | Borneo, Malaysia | LC056780 | Eto et al. (2015) |
| 44 | *Leptobrachella kajangensis* | LSUHC 4431 | Tioman, Malaysia | LC202001 | Matsui et al. (2017) |
| 45 | *Leptobrachella kalonensis* | AMNH A191762 | Binh Thuan, Vietnam | KR018115 | Rowley et al. (2015) |
| 46 | *Leptobrachella kecil* | KUHE 52440 | Cameron, Malaysia | LC202004 | Matsui et al. (2017) |
| 47 | *Leptobrachella khasiorum* | SDBDU 2009.329 | Khasi Hills, Meghalaya, India | KY022303 | Mahony et al. (2017) |
| 48 | *Leptobrachella korifi* | KUHE 19134 | Doi Inthanon, Chiang Mai, Thailand | LC741033 | Matsui et al. (2023) |
| 49 | *Leptobrachella lateralis* | IASST AR87 | India | KU674834 | Ao et al. (2023) |
| 50 | *Leptobrachella laui* | SYS a002450 | Shenzhen, Guangdong, China | MH055904 | Chen et al. (2018) |
| 51 | *Leptobrachella liui* | ZYCA907 | Wuyi Shan, Fujian, China | MH055908 | Chen et al. (2018) |
| 52 | *Leptobrachella macrops* | ZMMU-A5823 | Phu Yen, Vietnam | MG787993 | Duong et al. (2018) |
| 53 | *Leptobrachella maculosa* | AMS R 177660 | Ninh Thuan, Vietnam | KR018119 | Rowley et al. (2015) |
| 54 | *Leptobrachella mangshanensis* | MSZTC 201703 | Mangshan, Hunan, China | MG132198 | Hou et al. (2018) |
| 55 | *Leptobrachella maoershanensis* | KIZ 07614 | Mao’er Shan, Guangxi, China | MH055927 | Chen et al. (2018) |
| 56 | *Leptobrachella maoershanensis* | KIZ 027236 | Mao’er Shan, Guangxi, China | MH055928 | Chen et al. (2018) |
| 57 | *Leptobrachella marmorata* | KUHE53227 | Borneo, Malaysia | AB969289 | Matsui et al. (2014) |
| 58 | *Leptobrachella maura* | SP21450 | Borneo, Malaysia | AB847559 | Matsui et al. (2014) |
| 59 | *Leptobrachella melanoleucus* | KIZ018031 | Ranong, Thailand | MH055967 | Chen et al. (2018) |
| 60 | *Leptobrachella melica* | MVZ258198 | Cambodia, Ratanakiri | HM133600 | Rowley et al. (2010) |
| 61 | *Leptobrachella minima* | K3124 | Mae Lao Mae Sae, Doi Chiang Dao, Thailand | JN848369 | Ohler et al. (2011) |
| 62 | *Leptobrachella mjobergi* | KUHE:47872 | Borneo, Malaysia | LC056787 | Eto et al. (2015) |
| 63 | *Leptobrachella murphyi* | KIZ 034039 | Doi Inthanon, Chiang Mai, Thailand | MZ710519 | Chen et al. (2021) |
| 64 | *Leptobrachella nahangensis* | ROM 7035 | Na Hang Nature Reserve, Tuyen Quang, Vietnam | MH055853 | Chen et al. (2018) |
| 65 | *Leptobrachella namdongensis* | VNUF A.2017.37 | Thanh Hoa, Vietnam | MK965389 | Van Chung et al. (2019) |
| 66 | *Leptobrachella neangi* | CBC 1609 | Phnom Samkos Wildlife Sanctuary, Veal Veng District, Pursat, Cambodia | MT644613 | Stuart and Rowley (2020) |
| 67 | *Leptobrachella niveimontis* | KIZ 015734 | Daxueshan Nature Reserve, Yunnan, China | MT302618 | Chen et al. (2020) |
| 68 | *Leptobrachella nyx* | AMNH A163810 | Mount Tay Conn Linh, Ha Giang, Vietnam | DQ283381 | Frost et al. (2006) |
| 69 | *Leptobrachella nyx* | ROM 35606 | Malipo, Yunnan, China | MH055814 | Chen et al. (2018) |
| 70 | *Leptobrachella oshanensis* | KIZ 025776 | Emei Shan, Sichuan, China | MH055895 | Chen et al. (2018) |
| 71 | *Leptobrachella pallida* | UNS00511 | Vietnam, Lam Dong | KU530190 | Rowley et al. (2016) |
| 72 | *Leptobrachella parva* | KUHE:55308 | Sarawak, Malaysia | LC056791 | Eto et al. (2015) |
| 73 | *Leptobrachella pelodytoides* | ROM18282 | Vinh Phu, Vietnam | EF397244 | Fu et al. (2007) |
| 74 | *Leptobrachella petrops* | ROM 13483 | Ba Vi National Park, Ha Tay, Vietnam | MH055901 | Chen et al. (2018) |
| 75 | *Leptobrachella phiadenensis* | IEBR A.5206 | Phia Oac-Phia Den NP, Cao Bang, Vietnam | OR405873 | Luong et al. (2023) |
| 76 | *Leptobrachella picta* | UNIMAS 8705 | Borneo, Malaysia | KJ831295 | Oberhummer et al. (2014) |
| 77 | *Leptobrachella pluvialis* | MNHN 1999.5675 | Sapa, Vietnam | JN848391 | Ohler et al. (2011) |
| 78 | *Leptobrachella puhoatensis* | AMS R184852 | Pu Hoat Nature Reserve, Nghe An, Vietnam | KY849588 | Rowley et al. (2017) |
| 79 | *Leptobrachella purpuraventra* | SYS a007283 | Wujing Nature Reserve, Guizhou, China | MK414523 | Wang et al. (2019) |
| 80 | *Leptobrachella purpurus* | SYS a006531 | Jinzhuzhai Village, Yunnan, China | MG520355 | Yang et al. (2018) |
| 81 | *Leptobrachella pyrrhops* | ZMMU-A-4873-00158 | Lam Dong, Vietnam | MH055950 | Chen et al. (2018) |
| 82 | *Leptobrachella sabahmontana* | BORNEENSIS 12632 | Borneo, Malaysia | AB847551 | Matsui et al. (2014) |
| 83 | *Leptobrachella shangsiensis* | NHMG 1401032 | Guangxi, China | MK095460 | Chen et al. (2019) |
| 84 | *Leptobrachella shimentaina* | SYS a007684 | Shimentai Nature Reserve, Yingde City, Guangdong, China | ON017330 | Wang et al. (2022) |
| 85 | *Leptobrachella shiwandashanensis* | NNU 202103213 | Golden Camellia National Nature Reserve, Guangxi, China | MZ326692 | Chen et al. (2020) |
| 86 | *Leptobrachella sinorensis* | KUHE 19809 | Mae Hong Son, Thailand | LC741034 | Matsui et al. (2023) |
| 87 | *Leptobrachella sola* | KU RMB20973 | Malaysia | MH055973 | Chen et al. (2018) |
| 88 | *Leptobrachella suiyangensis* | GZNU 20180606002 | Suiyang County, Guizhou, China | MK829648 | Luo et al. (2020) |
| 89 | *Leptobrachella sungi* | ROM 20236 | Tam Dao, Vinh Phuc, Vietnam | MH055858 | Chen et al. (2018) |
| 90 | *Leptobrachella tadungensis* | UNS00515 | Dak Nong, Vietnam | KR018121 | Rowley et al. (2015) |
| 91 | *Leptobrachella tamdil* | MZMU2225 | NA | MW665131 | Unpublished |
| 92 | *Leptobrachella tengchongensis* | SYS a003766 | Gaoligong Shan, Tengchong County, Yunnan, China | MH055897 | Chen et al. (2018) |
| 93 | *Leptobrachella tuberosa* | ZMMU-NAP-02275 | Gia Lai,Vietnam | MH055959 | Chen et al. (2018) |
| 94 | *Leptobrachella ventripunctata* | KIZ 020421 | Jinuo Shan, Yunnan, China | MH055825 | Chen et al. (2018) |
| 95 | *Leptobrachella verrucosa* | GEP a062 | Lianshan Bijiashan Nature Reserve, Lianshan County, Guangdong, China | OP279592 | Lin et al. (2022) |
| 96 | *Leptobrachella wuhuangmontis* | SYS a003485 | Mt. Wuhuang, Pubei County, Guangxi, China | MH605577 | Wang et al. (2018) |
| 97 | *Leptobrachella wulingensis* | CSUFT 177 | Tianquanshan Forest Park, Zhangjiajie, Hunan, China | MT530315 | Qian et al. (2020) |
| 98 | *Leptobrachella wumingensis* | NNU 01058 | Wuming County, Guangxi, China | OR194551 | Chen et al. (2023) |
| 99 | *Leptobrachella xishuiensis* | GZNU20240726007 | Xianyuan Town, Xishui County, Guizhou, China | PQ604660 | Luo et al. (2025) |
| 100 | *Leptobrachella yeae* | CIB EMS20190422HLJ2-1 | Heilongjiang, Mount Emei, Sichuan, China | MT957022 | Shi et al. (2021) |
| 101 | *Leptobrachella yongshunensis* | HJ2024001 | Hunan Xiaoxi National Nature Reserve, Hunan Province, China | PQ227124 | Huang et al. (2025) |
| 102 | *Leptobrachella yingjiangensis* | SYS a006535 | Jinzhuzhai Village, Yunnan, China | MG520357 | Yang et al. (2018) |
| 103 | *Leptobrachella yunkaiensis* | SYS a004663 | Dawuling Forest Station, Guangdong, China | MH605584 | Wang et al. (2018) |
| 104 | *Leptobrachella yunkaiensis* | SYS a004665 | Dawuling Forest Station, Guangdong, China | MH605586 | Wang et al. (2018) |
| 105 | *Leptobrachella yunkaiensis* | SYS a004666 | Dawuling Forest Station, Guangdong, China | MH605587 | Wang et al. (2018) |
| 106 | *Leptobrachella yunkaiensis* | SYS a004667 | Dawuling Forest Station, Guangdong, China | MH605588 | Wang et al. (2018) |
| 107 | *Leptobrachella yunyangensis* | GZNU 20210629001 | Lianhua Village, Yunyang County, Chongqing, China | OL800366 | Luo et al. (2022) |
| 108 | *Leptobrachella zhangyapingi* | JK-2013 | Pang Num Poo, Chiang Mai, Thailand | JX069979 | Jiang et al. (2013) |
| 109 | *Oreolalax rhodostigmatus* | CIB ZYCA746 | Guizhou, China | EF397248 | Fu et al. (2007) |
| 110 | *Leptobrachium tengchongense* | SYS a004603 | Yunnan, China | KX066876 | Yang et al. (2016) |
